# Supplementary material for: Identification of genotype–biochemical phenotype correlations associated with fructose 1,6-bisphosphatase deficiency
Source: Commun Biol. 2023 Jul 28;6:787. doi: 10.1038/s42003-023-05160-y (PMC10382519; doi:10.1038/s42003-023-05160-y)
Supplement: Supplementary file 2 — Supplementary Information [file 42003_2023_5160_MOESM2_ESM.pdf]

**Supplementary Figure 1**

a

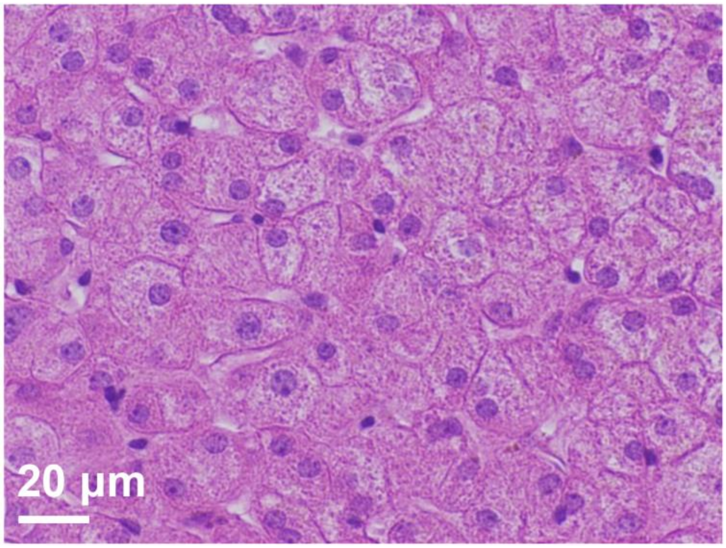


b

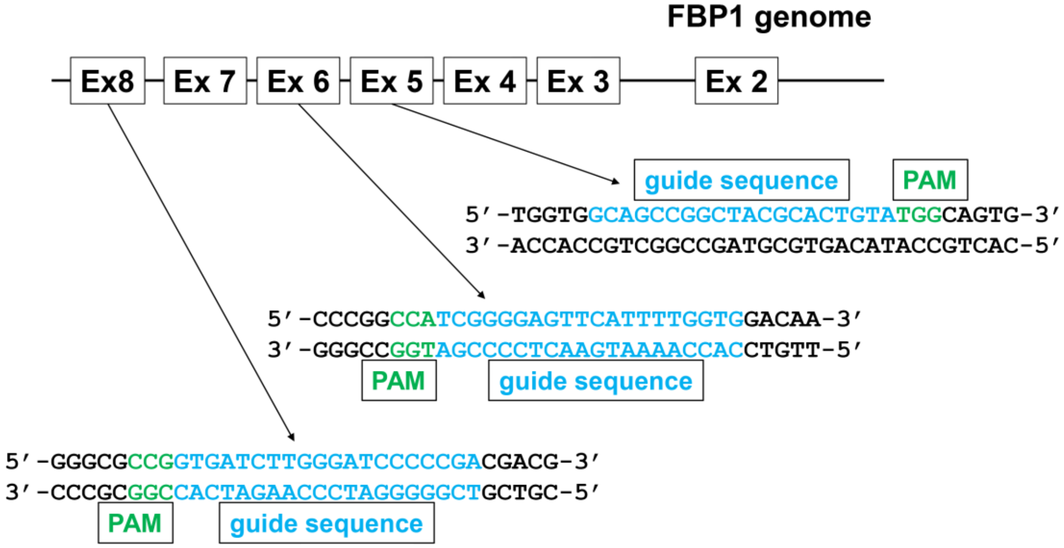


**a** Hematoxylin and eosin staining of a liver biopsy specimen obtained from the patient. The scale bar indicates 20 μm.

**b** Generation of *FBP1*-KO HepG2 cells using the CRISPR/Cas9 system. Guide sequences were designed at exon 5, exon 6, and exon 8.

**Supplementary Figure 2**

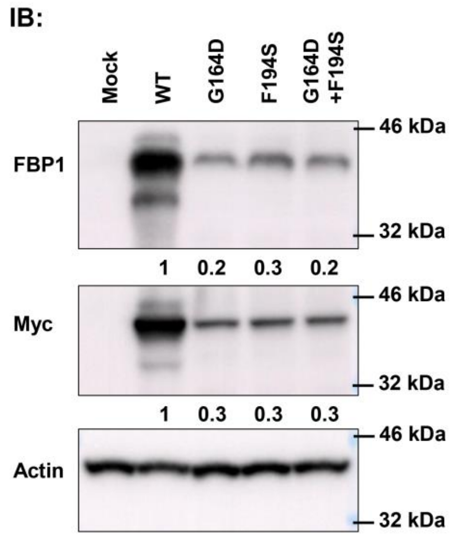


**Another immunoblots performed under the same conditions as Figure 2a using a CCD imager Fusion FX.**

Myc-tagged FBP1 and actin protein bands and size markers using Fusion FX were consistent with those labeled by a Sharpie marker using ECL-films in Figure 2a.

**Supplementary Figure 3**

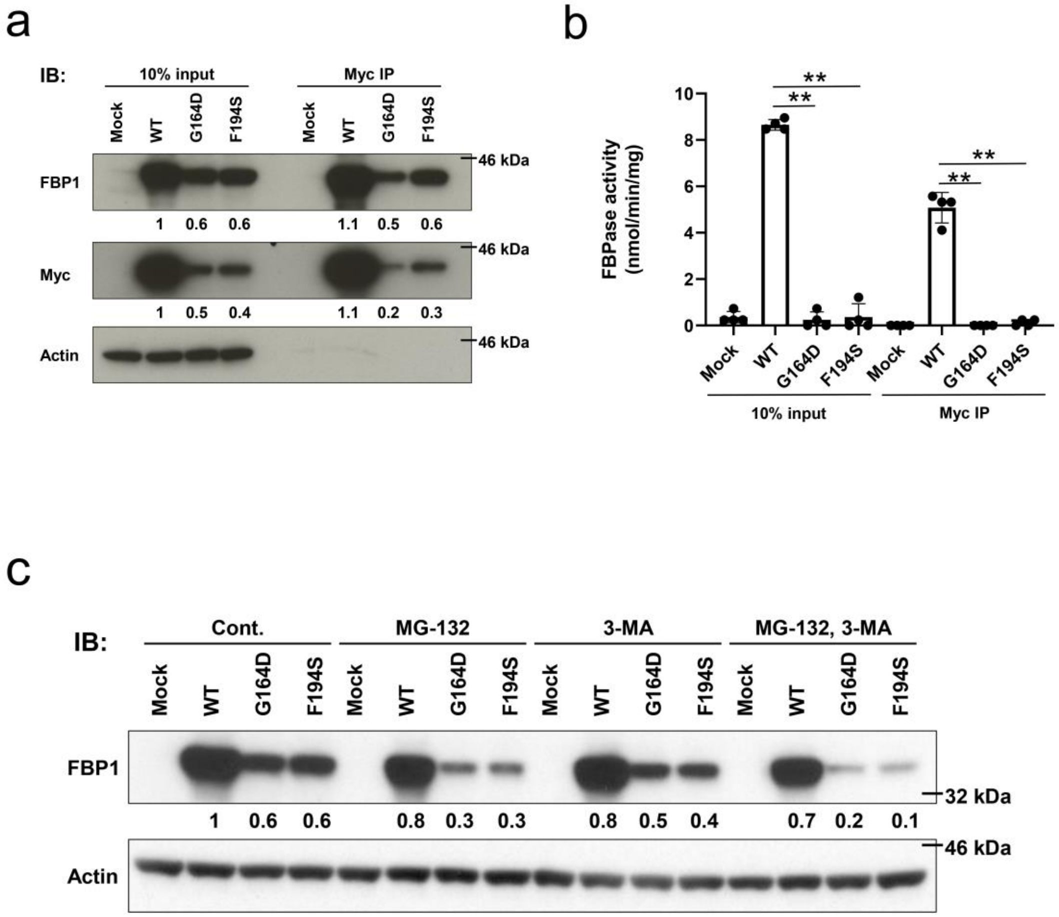


**The G164D and F194S FBP1 mutations cause a pathogenic loss of enzymatic activity and a decrease in protein expression.**

**a** Immunoprecipitation of WT FBP1 and the G164D and F194S mutants with anti-Myc antibodies, followed by immunoblot analysis and FBPase activity assay.

**b** Immunoprecipitated G164D and F194S showed no or little FBPase activity in NADP-coupled spectrophotometric assays. The data are presented as the mean ± SD. *$P < 0.05$; **$P < 0.01$ versus WT (one-way ANOVA test followed by Dunnett's multiple comparison test); n=4.

**c** HepG2 cells were treated with the proteasome inhibitor MG-132 (20 μM) and autophagy inhibitor 3-methyladenine (5 mM) for 24 h. Immunoblot analyses showed no restoration of the protein expression of the G164D and F194S FBP1 mutants.

Vertically stacked strips of bands in in **(a)** and **(c)** were evaluated in the same experimental conditions respectively while they were not in fact all derived from the same gel.

**Supplementary Figure 4**

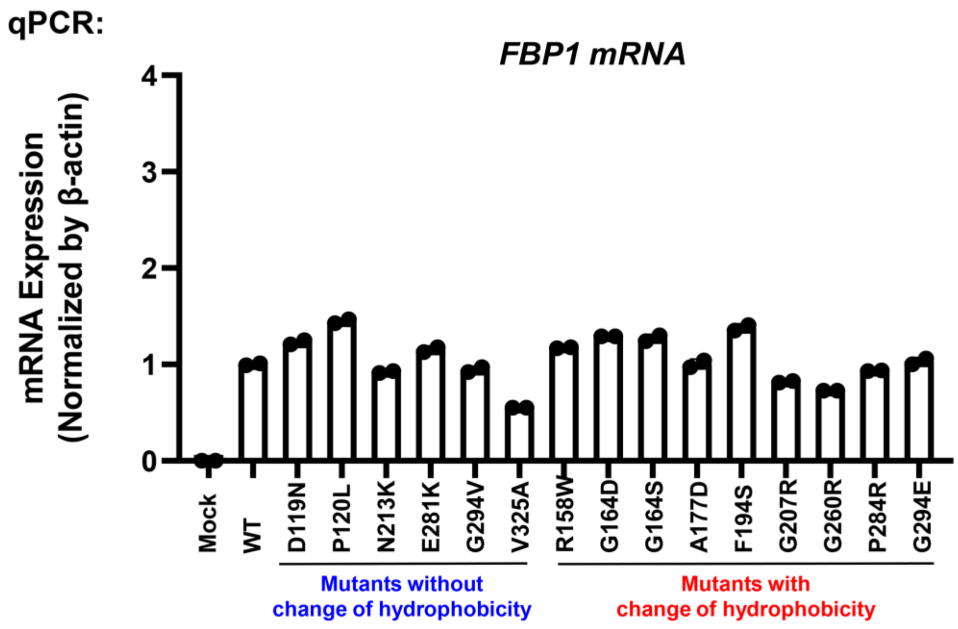


**mRNA expression of all previously reported FBP1 missense mutations.**

RT–qPCR analysis detected a sufficient amount of mRNA in the mutants. n=2

**Supplementary Figure 5**

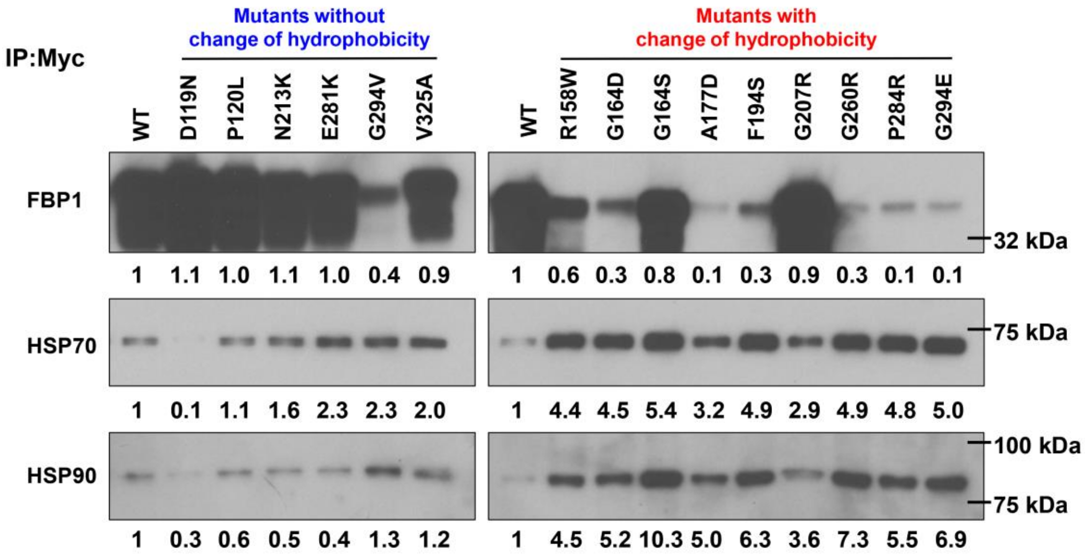


**Evaluation of the binding ability of HSP70 and HSP90 to FBP1 mutant proteins.**

Immunoprecipitation with anti-Myc followed by immunoblot analysis of FBP1, HSP70, and HSP90 showed that FBP1 mutants with changes in hydrophobicity exhibited greater interactions with HSP70 and HSP90 than wild-type (WT) FBP1.

Vertically stacked strips of bands in a figure were evaluated in the same experimental conditions respectively while they were not in fact all derived from the same gel.

# Supplementary Figure 6

## Uncropped Images

# Full unedited gel for Figure 2a

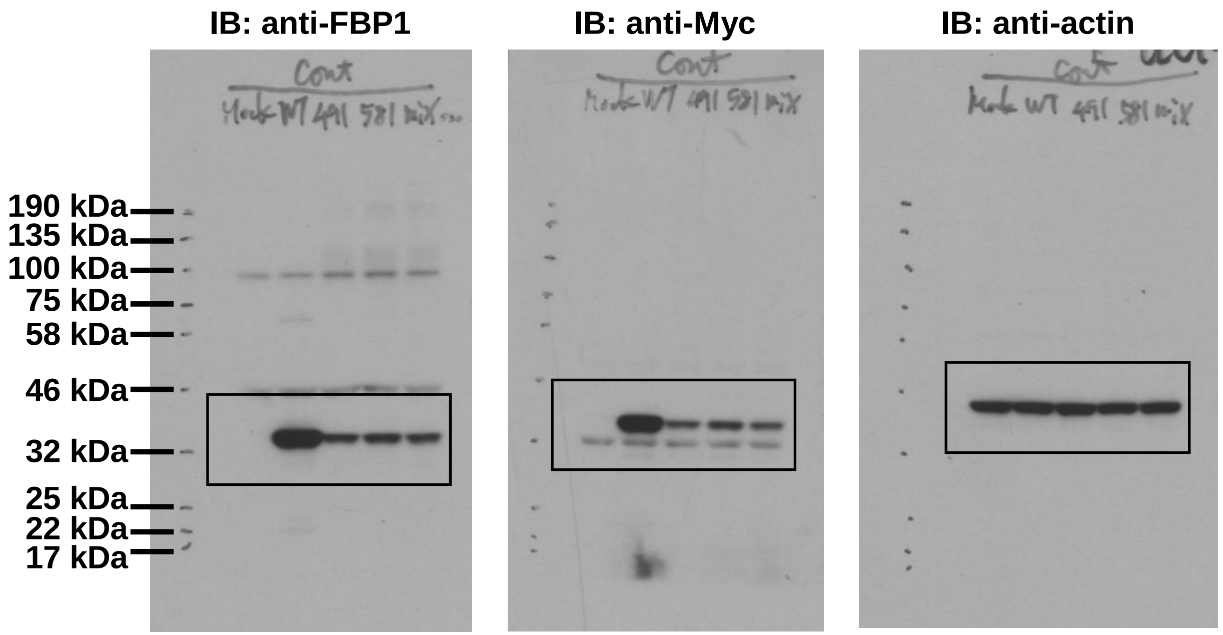

# Full unedited gel for Figure 2h

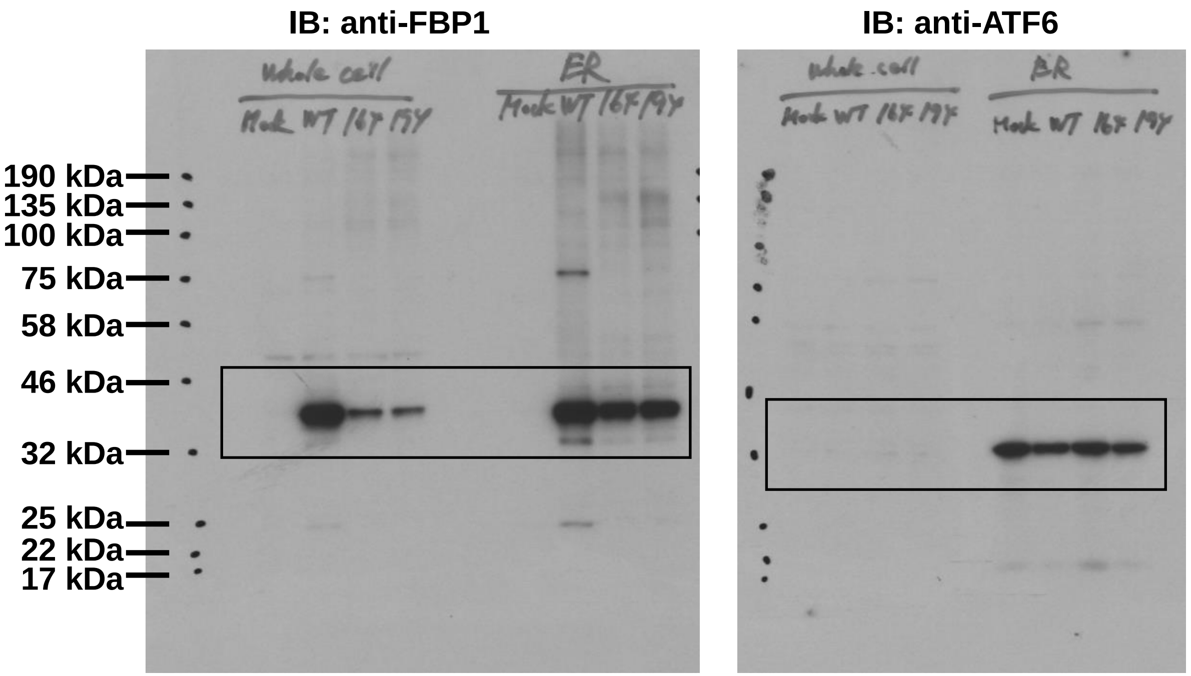

# Full unedited gel for Figure 2i

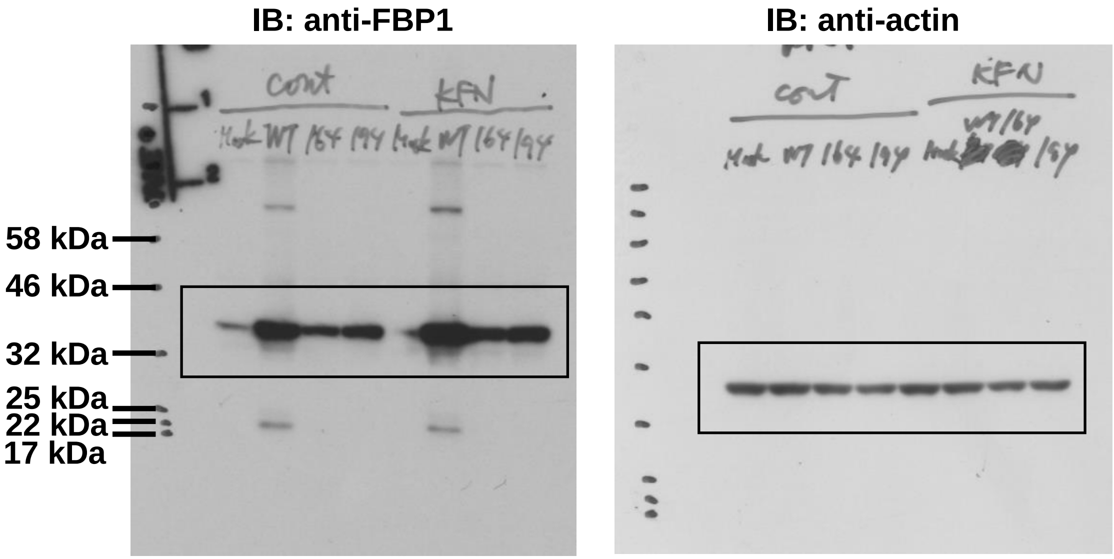

# Full unedited gel for Figure 2j

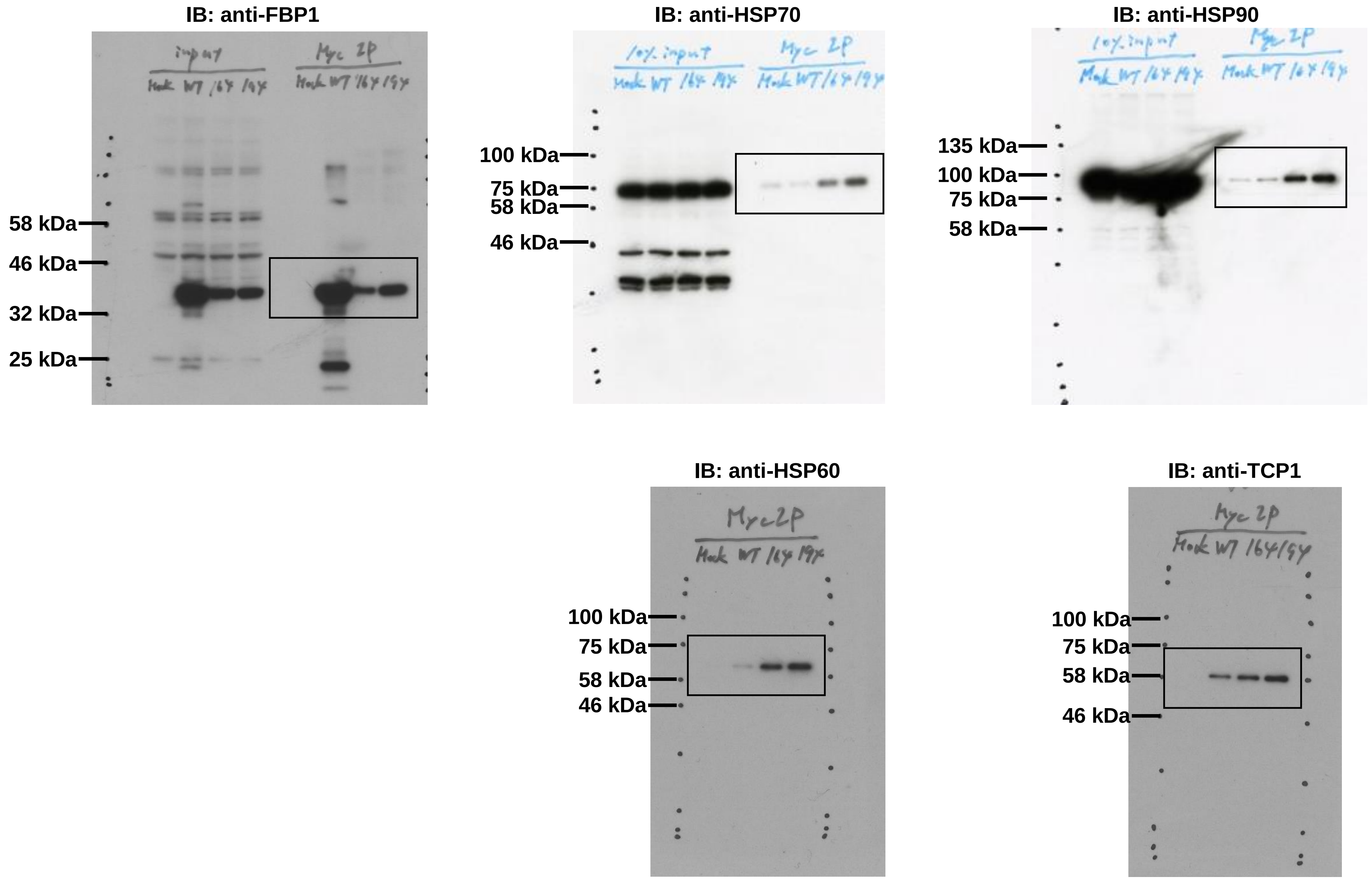

# Full unedited gel for Figure 3c

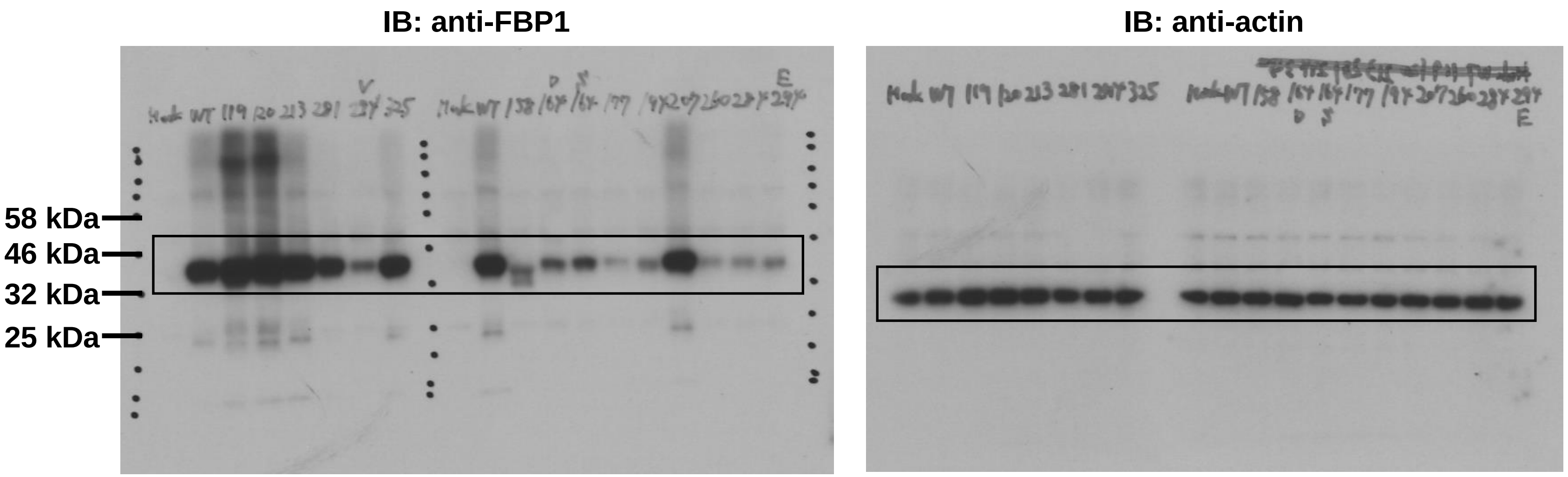

# Full unedited gel for Figure S2

IB: anti-FBP1

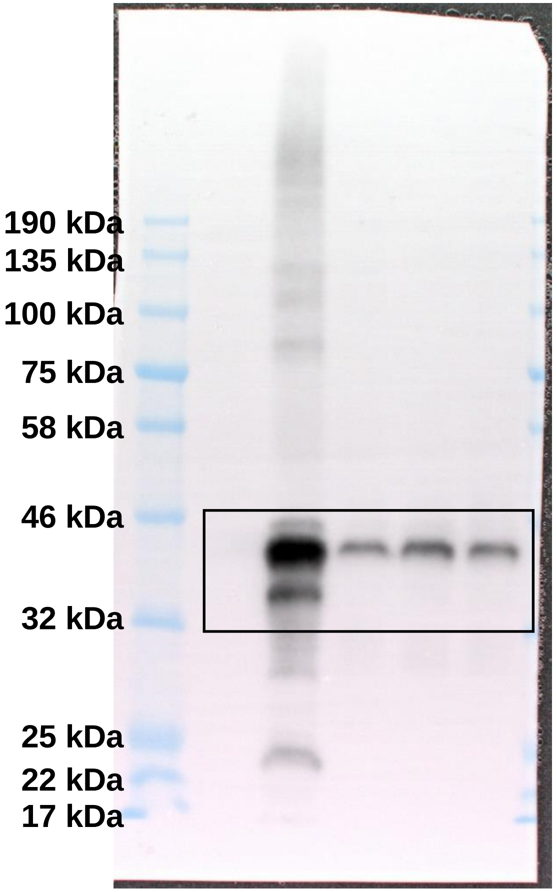


IB: anti-Myc

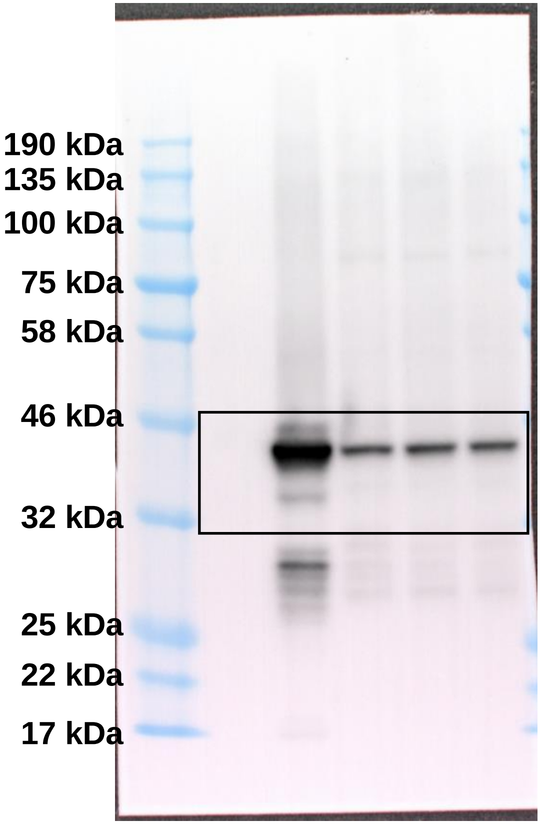


IB: anti-actin

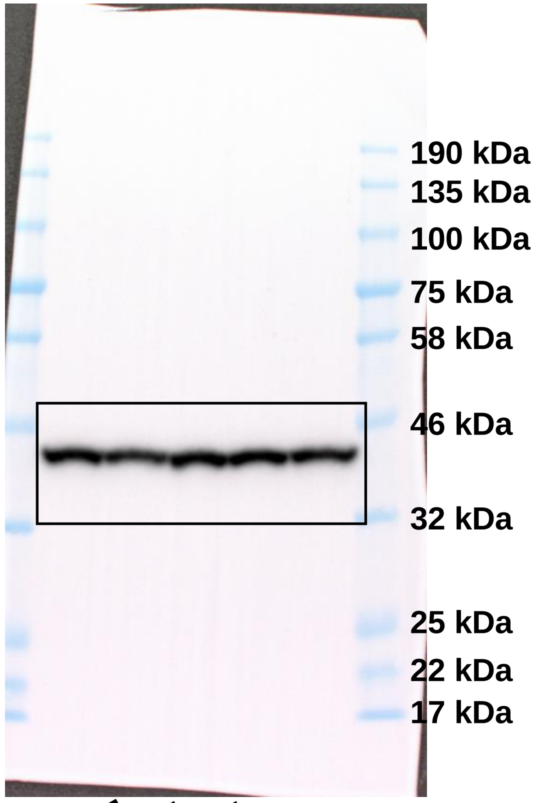

# Full unedited gel for Figure S3a

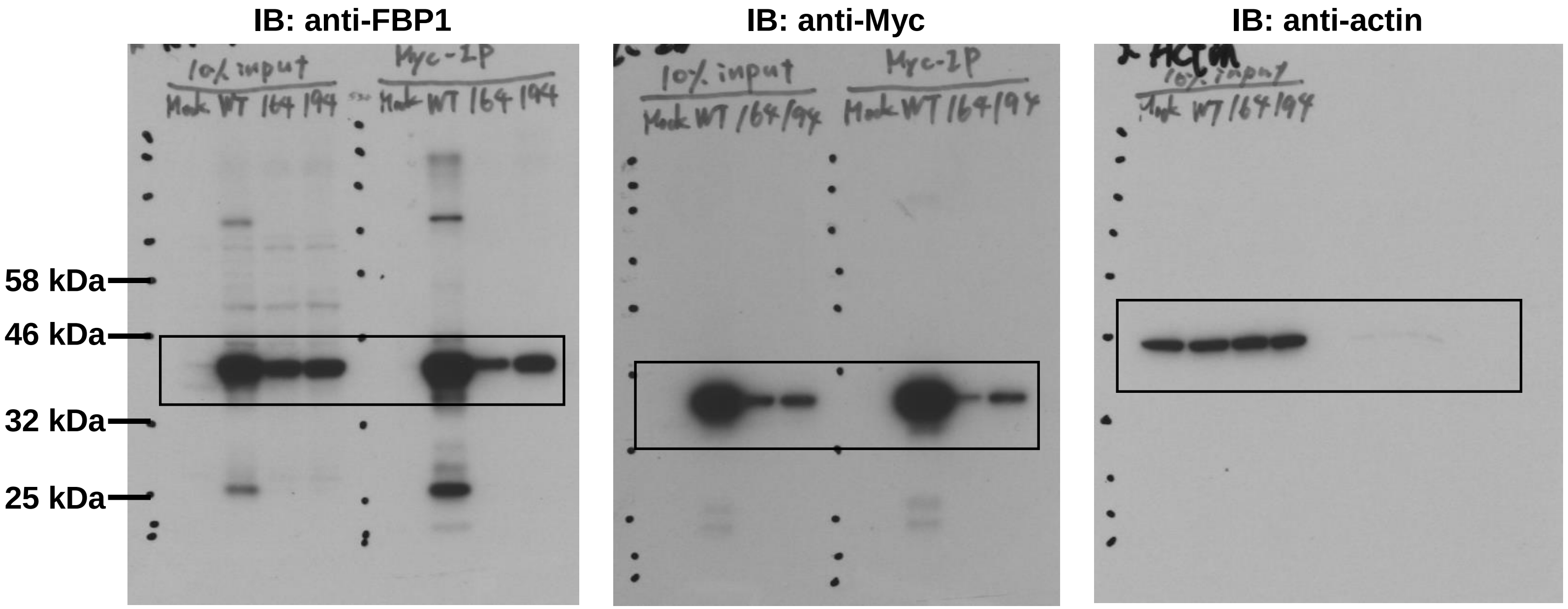

# Full unedited gel for Figure S3c

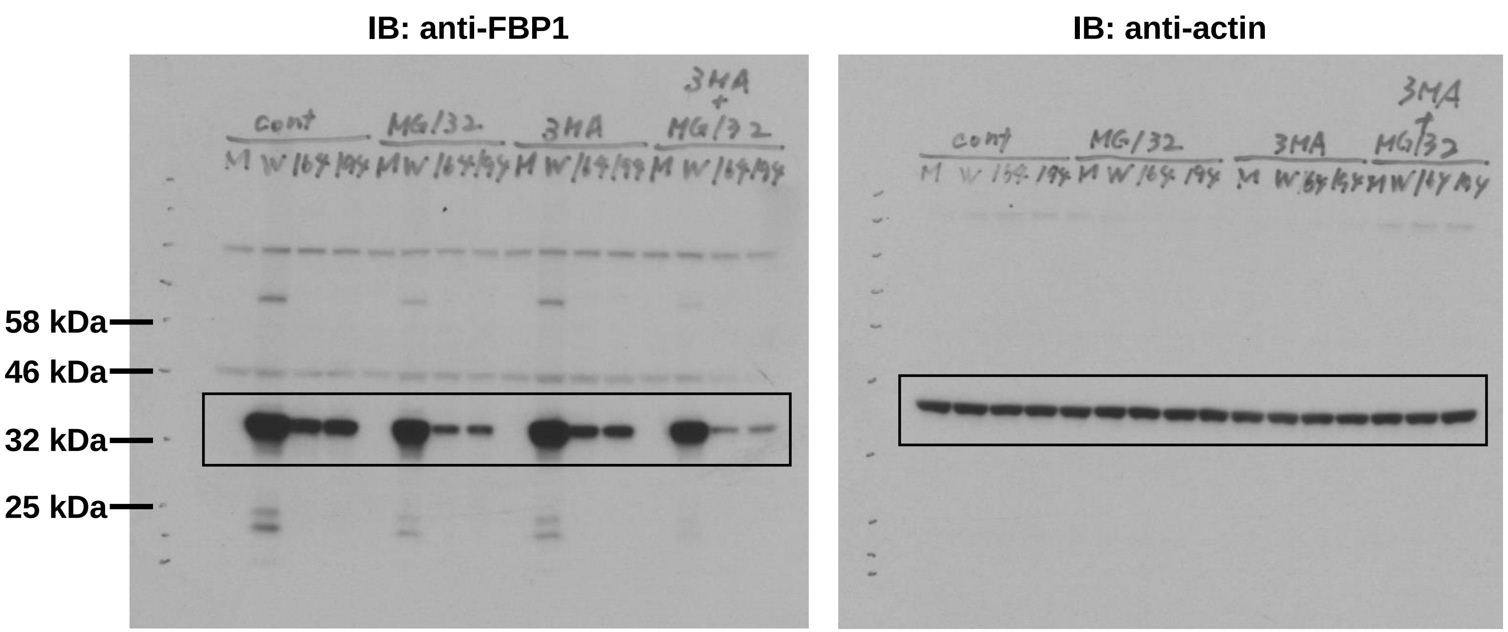

# Full unedited gel for Figure S5

**IB: anti-FBP1**

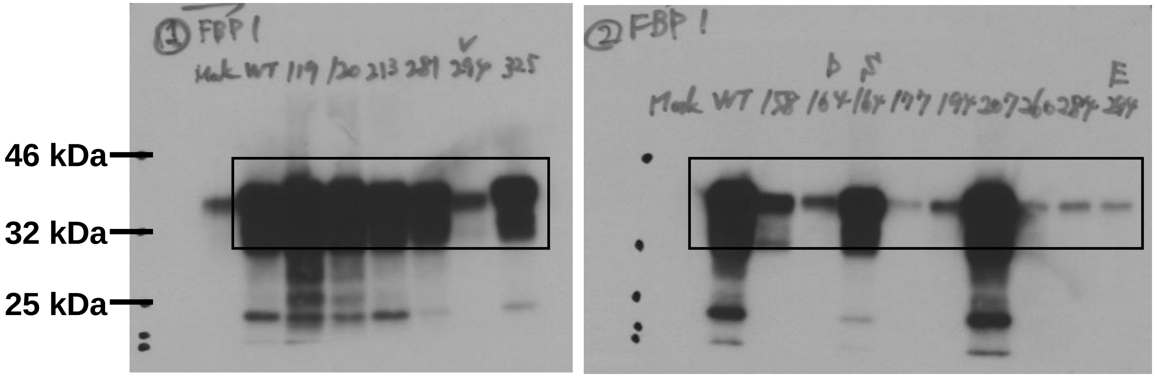


**IB: anti-HSP70**

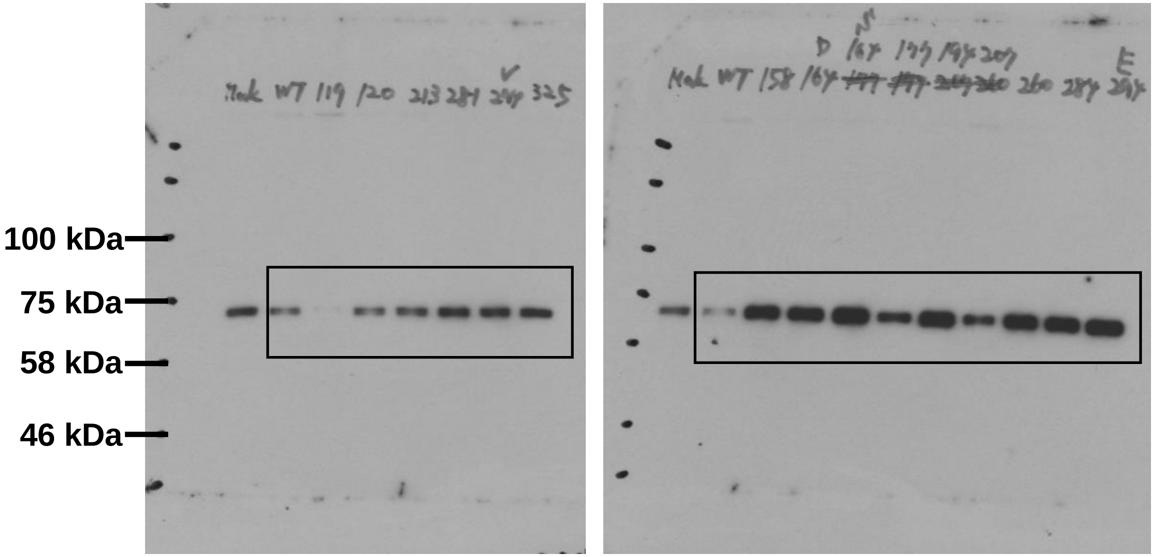


**IB: anti-HSP90**

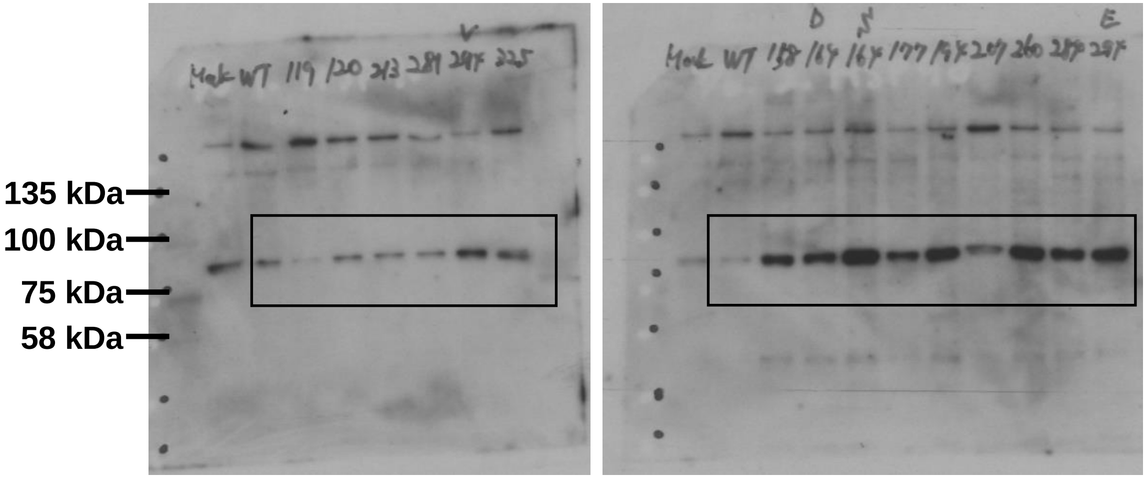

# Raw data for Figure 2d

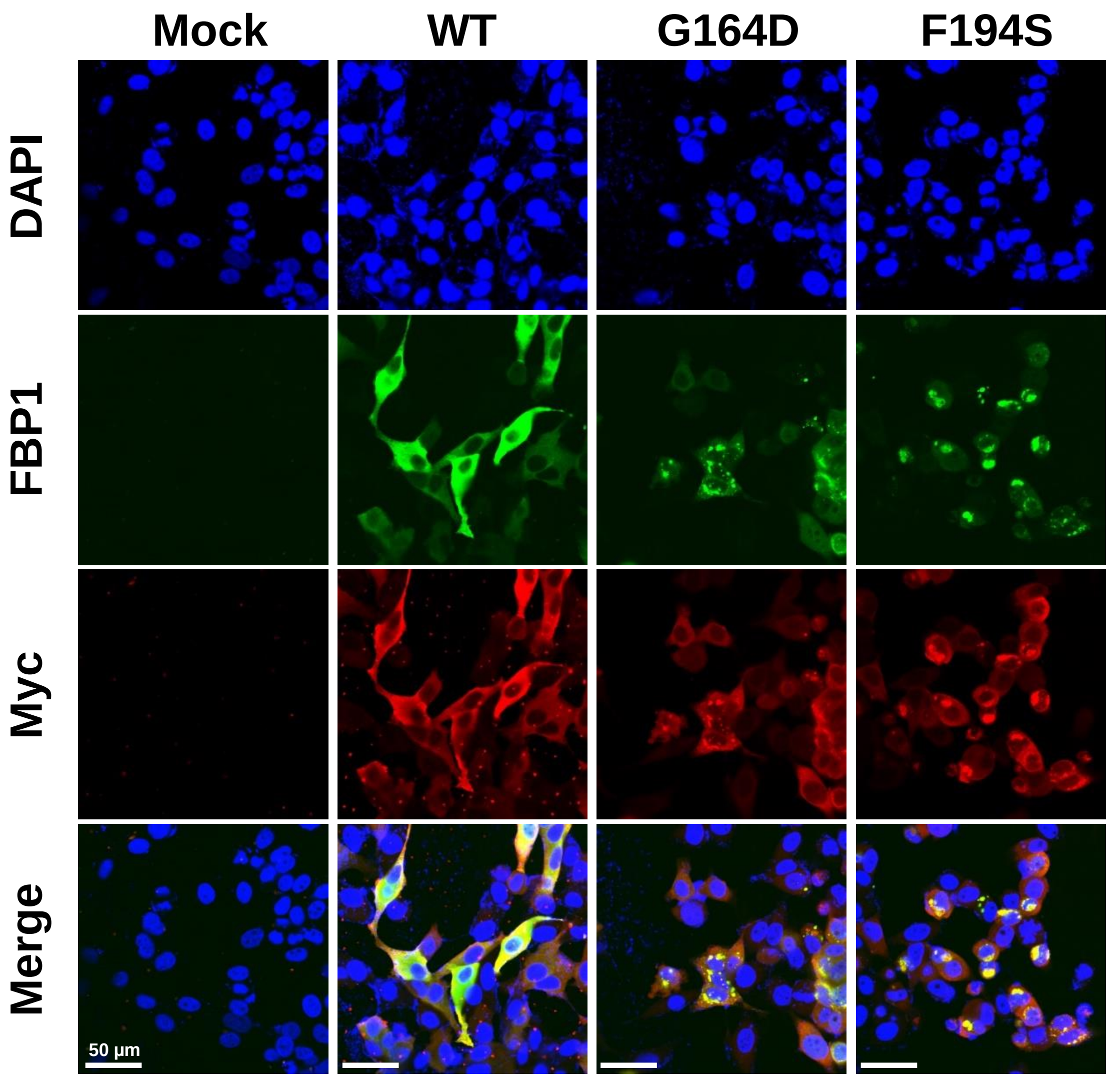

# Raw data for Figure 2f

FBP1

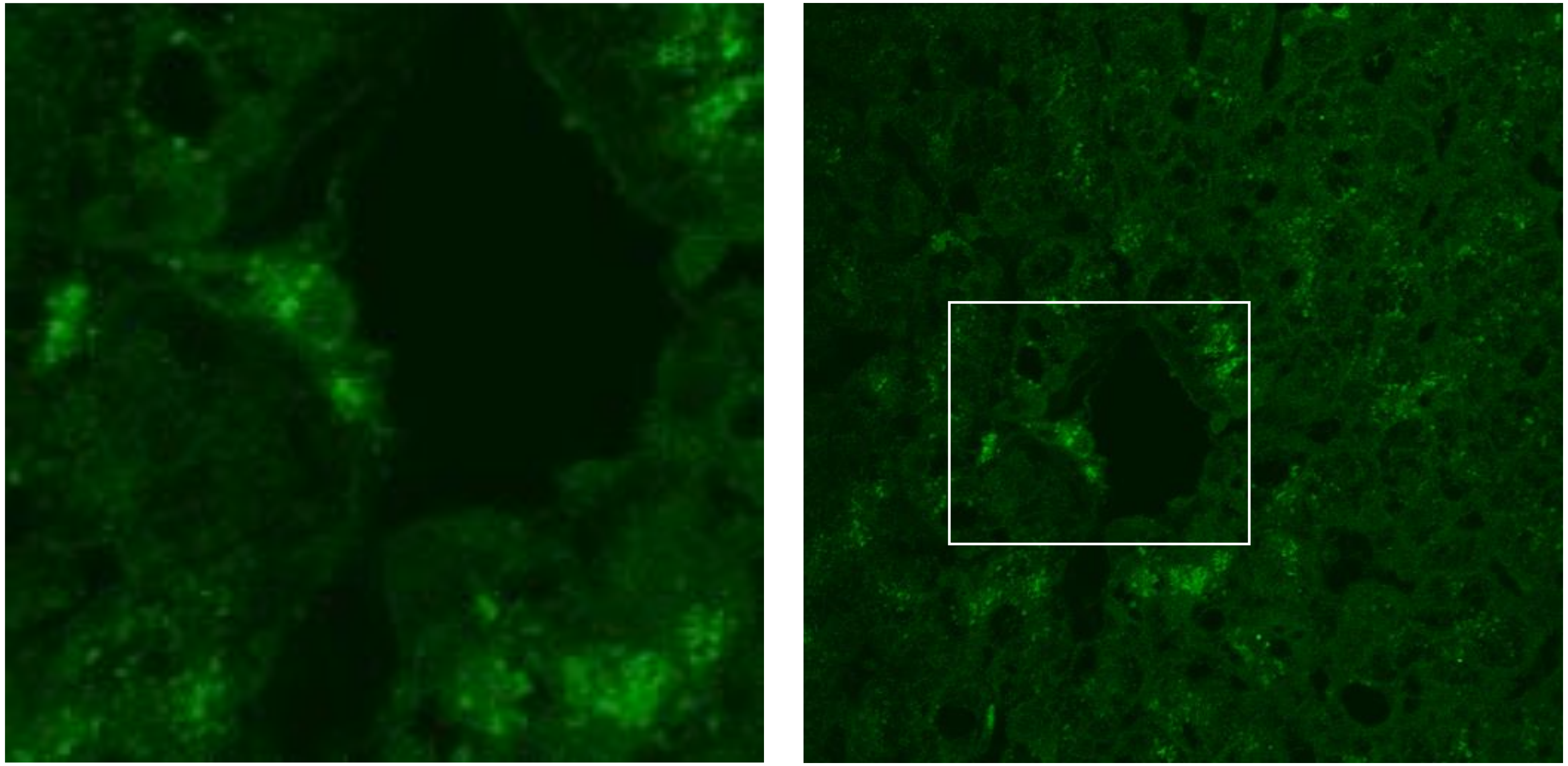

# Raw data for Figure 2f

Merge

FBP1+DAPI

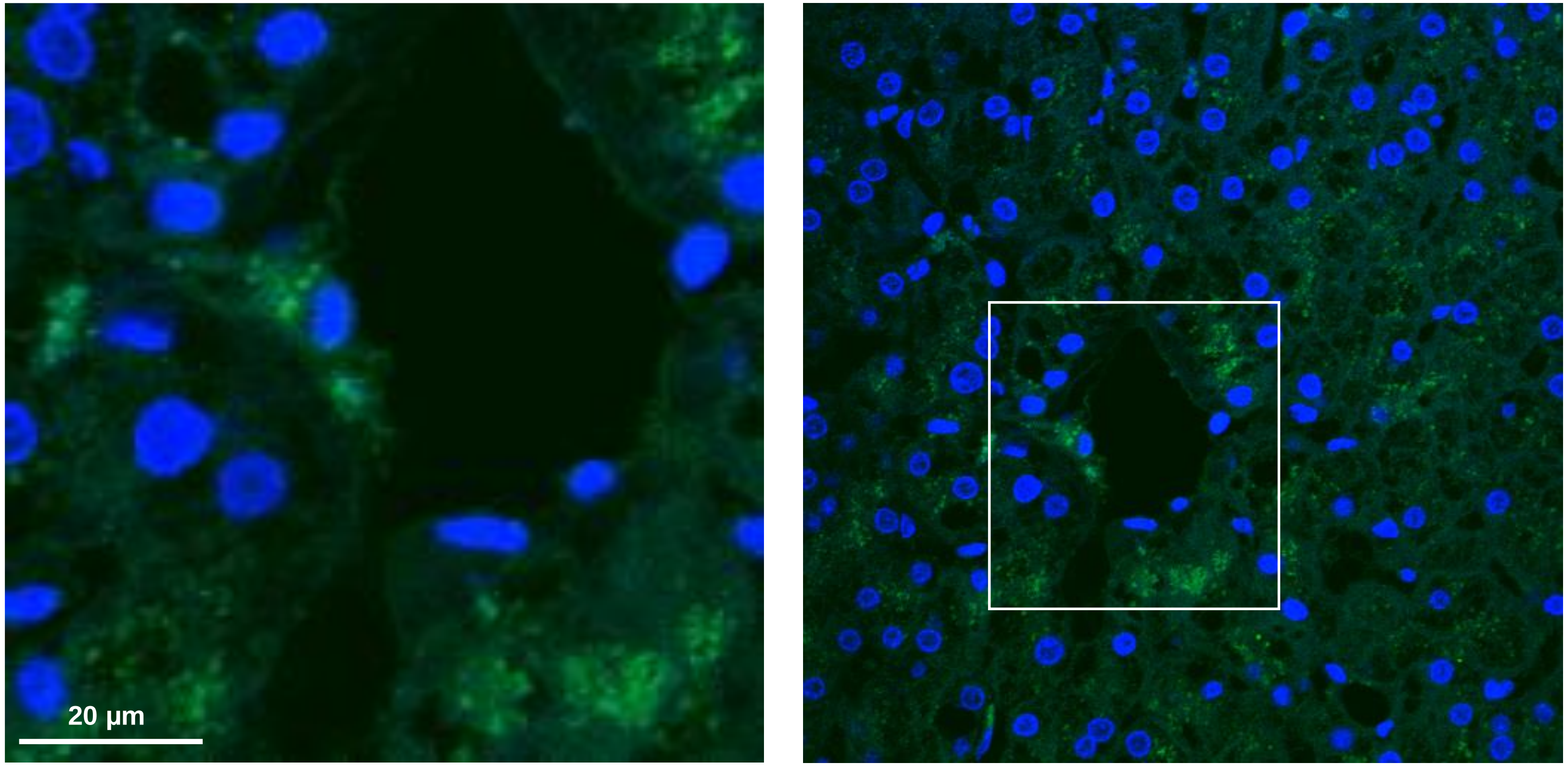

# Raw data for Figure 2g

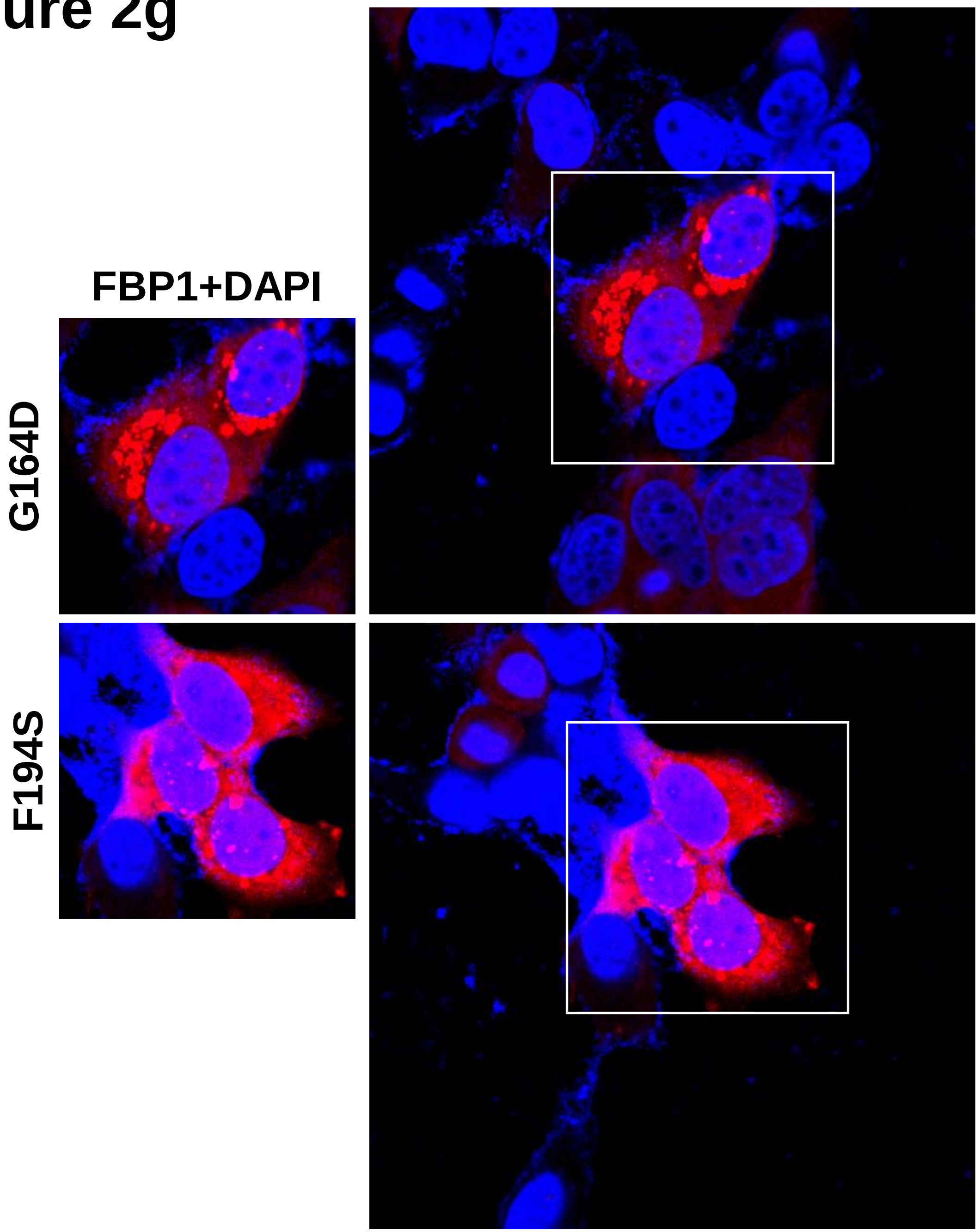

# Raw data for Figure 2g

E
R

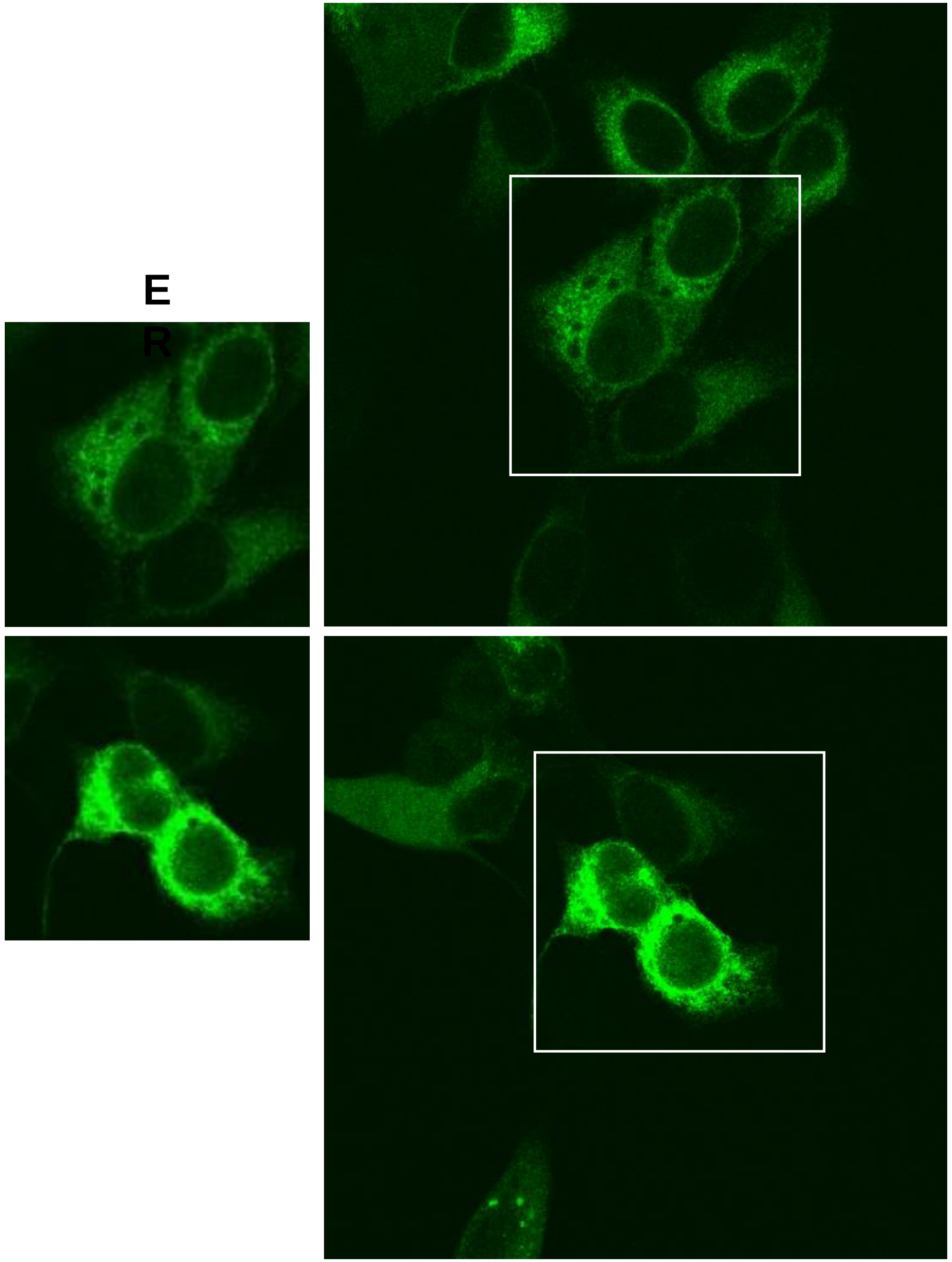

# Raw data for Figure 2g

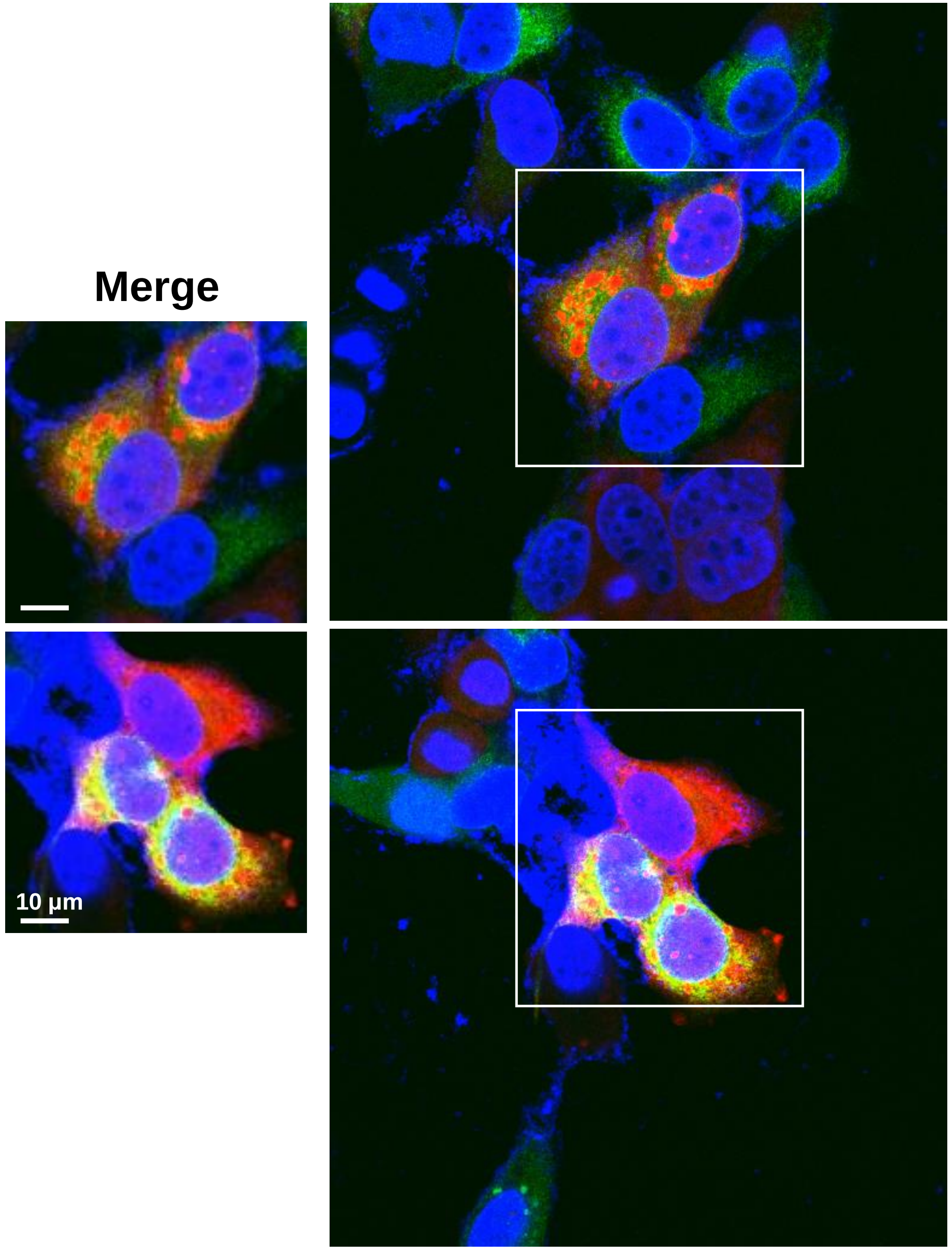

# Raw data for Figure 4a

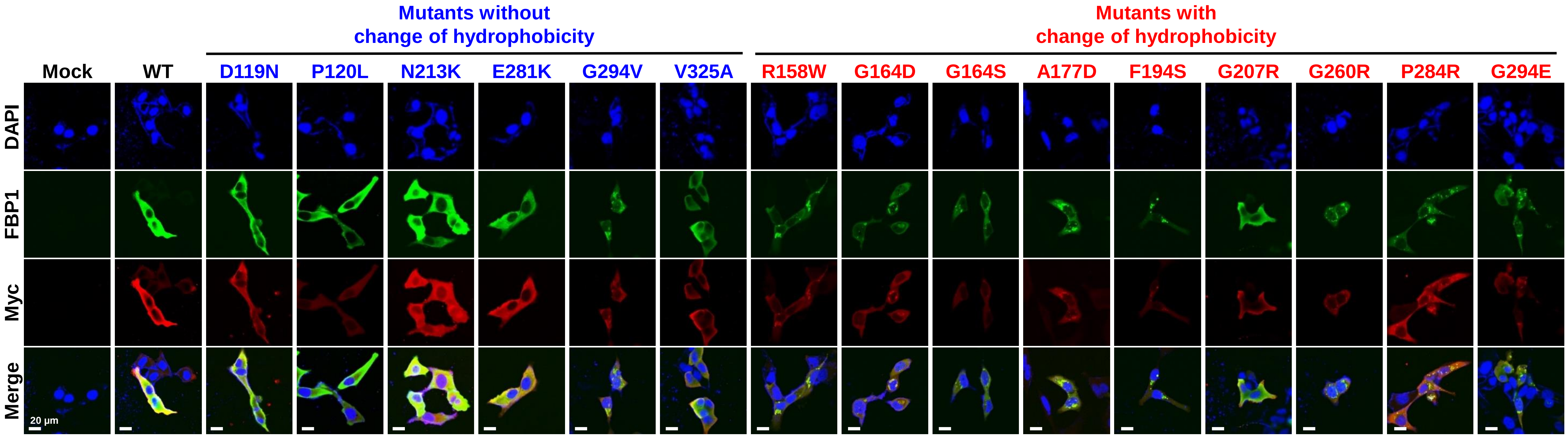

# Raw data for FigureS1a

HE

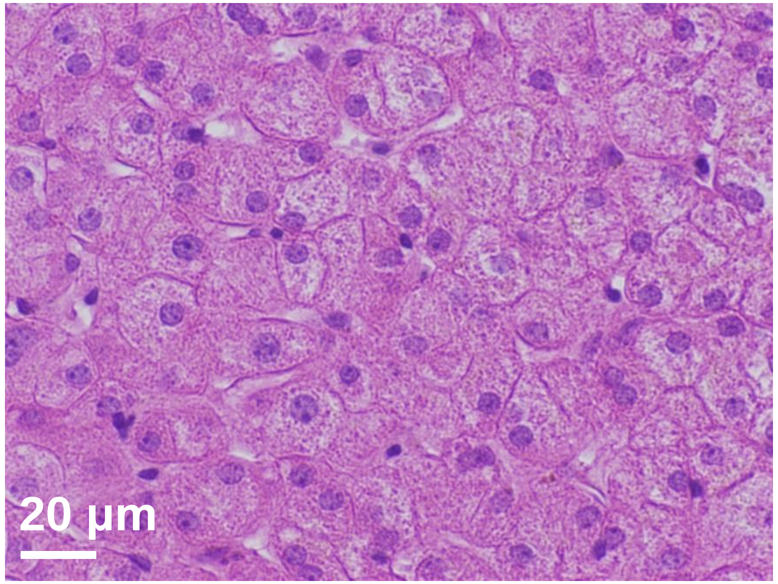

**Supplementary Table 1. Profiles of blood metabolites during several attacks in the present case.**

| Age | Trigger | Glucose mg/dL (70-109) | Lactate mg/dL (3.0-17.0) | 3OHB µmol/L (<85) | FFA µEq/L (140-850) | 3OHB/FFA ratio (>1.0) |
|---|---|---|---|---|---|---|
| 3 | Prolonged fasting | 4 | 57 | ND | ND | ND |
| 22 | Prolonged fasting under stress and fatigue conditions | 12 | 111 | 303 | 698 | 0.43 |
| 22 | Prolonged fasting with fructose loading | 29 | 54 | 354 | 3278 | 0.11 |

Abbreviations: 3OHB, 3-hydroxybutyrate; FFA, free fatty acid; ND, no data

## Supplementary Table 2. List of proteins identified by LC-MS/MS

| Function | Description | Mascot Protein Score | | | Fold change | |
|---|---|---|---|---|---|---|
| | | WT | G164D | F194S | G164D/WT | F194S/WT |
| unfolded protein binding | TCP1 | 135 | 329 | 404 | 2.4 | 3.0 |
| unfolded protein binding | CCT7 | 289 | 636 | 520 | 2.2 | 1.8 |
| unfolded protein binding | CCT3 | 276 | 603 | 618 | 2.2 | 2.2 |
| unfolded protein binding | CCT6A | 185 | 322 | 308 | 1.7 | 1.7 |
| unfolded protein binding | TUBB4B | 200 | 347 | 432 | 1.7 | 2.2 |
| unfolded protein binding | NPM1 | 158 | 245 | 227 | 1.6 | 1.4 |
| unfolded protein binding | HSPD1 | 390 | 587 | 749 | 1.5 | 1.9 |
| unfolded protein binding | HSPA9 | 410 | 613 | 492 | 1.5 | 1.2 |
| unfolded protein binding | CCT4 | 438 | 570 | 543 | 1.3 | 1.2 |
| unfolded protein binding | PPIB | 156 | 189 | 247 | 1.2 | 1.6 |
| unfolded protein binding | HSPA5 | 653 | 763 | 954 | 1.2 | 1.5 |
| heat shock protein binding | TPR | 83 | 291 | 484 | 3.5 | 5.8 |
| heat shock protein binding | HSPA8 | 492 | 910 | 1187 | 1.8 | 2.4 |
| heat shock protein binding | DNAJC7 | 72 | 126 | 84 | 1.8 | 1.2 |
| heat shock protein binding | DNAJB6 | 72 | 52 | 108 | 0.7 | 1.5 |
| Protein processing in endoplasmic reticulum | SEC23B | 75 | 212 | 181 | 2.8 | 2.4 |
| Protein processing in endoplasmic reticulum | HSP90B1 | 126 | 343 | 344 | 2.7 | 2.7 |
| Protein processing in endoplasmic reticulum | HSP90AA1 | 129 | 315 | 265 | 2.4 | 2.1 |
| Protein processing in endoplasmic reticulum | SEC23A | 61 | 148 | 157 | 2.4 | 2.6 |
| Protein processing in endoplasmic reticulum | BAG2 | 44 | 93 | 109 | 2.1 | 2.5 |
| Protein processing in endoplasmic reticulum | EIF2S1 | 78 | 158 | 200 | 2.0 | 2.6 |
| Protein processing in endoplasmic reticulum | HSP90AB1 | 276 | 494 | 417 | 1.8 | 1.5 |
| Protein processing in endoplasmic reticulum | P4HB | 142 | 208 | 277 | 1.5 | 2.0 |
| Protein processing in endoplasmic reticulum | SEC13 | 170 | 229 | 170 | 1.3 | 1.0 |
| Protein processing in endoplasmic reticulum | SEC24C | 0 | 0 | 178 | | |
| Protein processing in endoplasmic reticulum | DNAJB11 | 0 | 0 | 39 | | |
| Protein processing in endoplasmic reticulum | PDIA3 | 51 | 0 | 0 | 0.0 | 0.0 |
| Proteasome | PSMD2 | 40 | 137 | 128 | 3.4 | 3.2 |
| Proteasome | PSMD3 | 49 | 103 | 131 | 2.1 | 2.7 |
| Proteasome | PSME3 | 0 | 91 | 103 | | |

**Supplementary Table 3. Primers used to genotype FBP1 and PCR conditions.**

| | | Primers | PCR product size (bp) | Annealing temp. (°C) |
|---|---|---|---|---|
| Exon 2 | Forward | GACCCGCGTCTAAAGGTTTC | 504 | 55 |
| | Reverse | AGAGGGCCCAACGTCAG | | |
| Exon 3 | Forward | TGGACTCATCTACATGTTCTGG | 294 | 52 |
| | Reverse | GGAGTCAATTATGAAGCTGGG | | |
| Exon 4 | Forward | AAATTCCCATTCTTAGCCCC | 257 | 55 |
| | Reverse | GCCACAGTGAAATAGGACTGG | | |
| Exon 5 | Forward | GAGAATGCCTCCTGTTAATG | 390 | 58 |
| | Reverse | CACTCTCTCTTGGTCTCCTG | | |
| Exon 6 | Forward | ATTCACAGTTCCCAGAGAGC | 508 | 58 |
| | Reverse | AATCCACTCCATCCCTGCTT | | |
| Exon 7 | Forward | TTAGGAGACACCAAGAAAACTCTC | 257 | 53 |
| | Reverse | CTAAACTAAATCGCGTGGGC | | |
| Exon 8 | Forward | ACTTTTACAGCCTCACAGGA | 404 | 58 |
| | Reverse | GGTACTGCTGTGTGAGACAA | | |

PCR conditions: 94°C for 2 min, followed by 30 cycles of 15 sec at 94°C, each appropriate annealing temperature for 30 sec, 72°C for 45 sec, and 72°C for 7 min.

**Supplementary Table 4. Primers used to generate the FBP1 mutants.**

| Mutation | | Primers |
|---|---|---|
| D119N | Forward | AGGGGTAAATATGTGGTCTGTTTTAATCCCCTTGATGG |
| | Reverse | CCATCAAGGGGATTAAAACAGACCACATATTTACCCCT |
| P120L | Forward | TATGTGGTCTGTTTTGATCTCCTTGATGGATCTTCCAAC |
| | Reverse | GTTGGAAGATCCATCAAGGAGATCAAAACAGACCACATA |
| R158W | Forward | TCTGCAACCAGGCTGGAACCTGGTGGC |
| | Reverse | GCCACCAGGTTCCAGCCTGGTTGCAGA |
| G164S | Forward | CCTGGTGGCAGCCAGCTACGCACTGTA |
| | Reverse | TACAGTGCGTAGCTGGCTGCCACCAGG |
| A177D | Forward | CATGCTGGTCCTTGACATGGACTGTGGGG |
| | Reverse | CCCCACAGTCCATGTCAAGGACCAGCATG |
| G207R | Forward | GACAAGGATGTGAAGATAAAAAAGAAACGTAAAATCTACAGCCTTAAC |
| | Reverse | GTTAAGGCTGTAGATTTTACGTTTCTTTTTTATCTTCACATCCTTGTC |
| N213K | Forward | AAGGTAAAATCTACAGCCTTAAGGAGGGCTACGCC |
| | Reverse | GGCGTAGCCCTCCTTAAGGCTGTAGATTTTACCTT |
| G260R | Forward | ATCGCACTCTGGTCTACAGAGGGATATTTCTGTAC |
| | Reverse | GTACAGAAATATCCCTCTGTAGACCAGAGTGCGAT |
| E281K | Forward | GCTGAGACTGCTGTACAAATGCAACCCCATGGC |
| | Reverse | GCCATGGGGTTGCATTTGTACAGCAGTCTCAGC |
| P284R | Forward | CTGTACGAATGCAACCGCATGGCCTACGTCATG |
| | Reverse | CATGACGTAGGCCATGCGGTTGCATTCGTACAG |
| G294E | Forward | GGAGAAGGCTGGGGAAATGGCCACCACTG |
| | Reverse | CAGTGGTGGCCATTTCCCCAGCCTTCTCC |
| G294V | Forward | GGAGAAGGCTGGGGTAATGGCCACCACTG |
| | Reverse | CAGTGGTGGCCATTACCCCAGCCTTCTCC |
| V325A | Forward | CCCCCGACGACGCGCTCGAGTTCCT |
| | Reverse | AGGAACTCGAGCGCGTCGTCGGGGG |

**Supplementary Table 5. Oligos used to generate the guide RNA expression vectors.**

| FBP1 | | Oligos |
|---|---|---|
| Exon 5 | Forward | CACCGCAGCCGGCTACGCACTGTA |
| | Reverse | AAACTACAGTGCGTAGCCGGCTGC |
| Exon 6 | Forward | CACCGCACCAAAATGAACTCCCCGA |
| | Reverse | AAACTCGGGGAGTTCATTTTGGTGC |
| Exon 8 | Forward | CACCGTCGGGGGATCCCAAGATCAC |
| | Reverse | AAACGTGATCTTGGGATCCCCCGAC |

**Supplementary Table 6. Primers used for the real-time RT-PCR analysis.**

| Gene | | Primers | PCR product size (bp) |
|---|---|---|---|
| *FBP1* | Forward | GGAGGGATATTTCTGTACCCCGCTAA | 165 |
| | Reverse | CCTCTGGTGAATGTCTGTGGGAATGA | |
| *β-actin* | Forward | CCTGGCACCCAGCACA | 70 |
| | Reverse | GCCGATCCACACGGAG | |
