# Supplementary material for: Identification of genotype–biochemical phenotype correlations associated with fructose 1,6-bisphosphatase deficiency
Source: Commun Biol. 2023 Jul 28;6:787. doi: 10.1038/s42003-023-05160-y (PMC10382519; doi:10.1038/s42003-023-05160-y)
Supplement: Supplementary file 3 — Description of Additional Supplementary Files [file 42003_2023_5160_MOESM3_ESM.pdf]

Description of Additional Supplementary Files

File name: Supplementary Data 1

Description: QC of whole exome sequencing

File name: Supplementary Data 2

Description: Mass spectrometry data

File name: Supplementary Data 3

Description: The source data behind the graphs in the paper

File name: Supplementary Data 4

Description: Mutations detected by whole-exome sequencing
